# A new index for the outcome of focal segmental glomerulosclerosis

**DOI:** 10.1038/s41598-024-59007-5

**Published:** 2024-04-09

**Authors:** Liu Chan, Yang Danyi, Chao Chen

**Affiliations:** 1 Department of Nephrology, The Second Xiangya Hospital, Central South University, No.139, Renmin Road, Changsha, 410011 Hunan People’s Republic of China; 2 Hunan Key Laboratory of Kidney Disease and Blood, The Second Xiangya Hospital, Central South University, No.139, Renmin Road, Changsha, 410011 Hunan People’s Republic of China; 3 International Medical Department, The Second Xiangya Hospital, Central South University, Changsha, Hunan People’s Republic of China; 4 National Clinical Research Center for Metabolic Diseases, Key Laboratory of Diabetes Immunology, Ministry of Education, and Department of Metabolism and Endocrinology, The Second Xiangya Hospital of Central South University, Changsha, 410011 Hunan People’s Republic of China

**Keywords:** FSGS, Segmental glomerulosclerosis, Proportion, Podocyte, Outcome, Diseases, Medical research, Nephrology, Urology

## Abstract

Focal segmental glomerulosclerosis (FSGS) is a common pathological form of nephrotic syndrome. This study analyzed the value of pathological lesions and clinical prognosis of different segmental glomerulosclerosis ratios in FSGS. Two hundred and six FSGS patients were collected from Dec 2013 to Apr 2016. The patients were divided into two groups according to the proportion of glomerular segmental sclerosis: F1 (SSR ≤ 15%, n = 133) and F2 (SSR > 15%, n = 73). The clinical and pathological data were recorded and analyzed, and statistical differences were observed between the serum uric acid level and the percentage of chronic renal failure. The pathological results showed significant differences in interstitial fibrosis and tubular atrophy (IFTA), degree of mesangial hyperplasia, vascular lesions, synaptopodin intensity, and foot process effacement between the two groups. Multivariate logistic regression analysis showed significant differences in creatinine (OR: 1.008) and F2 group (OR: 1.19). In all patients, the prognoses of urine protein and serum creatinine levels were statistically different. Multivariate Cox regression analysis revealed that F2 (hazard ratio: 2.306, 95% CI 1.022–5.207) was associated with a risk of ESRD (end stage renal disease). The proportion of segmental glomerulosclerosis provides a guiding value in the pathological diagnosis and clinical prognosis of FSGS.

## Introduction

Focal segmental glomerulosclerosis (FSGS) is a common cause of nephrotic syndrome (NS) in adults. Approximately 20% of patients may eventually progress to end-stage renal disease (ESRD) due to its complex etiology, unclear pathogenesis, and insensitivity to glucocorticoid therapy. FSGS is a pathologic kidney diagnosis characterized by segmental scar sclerosis with or without glomerular foam cell formation and adhesion in capillaries^1^. In 1957, Rich found segmental glomerular sclerosis in the juxtamedullary region of a patient with lipoid nephrosis in the autopsy and presented FSGS as a pathomorphism diagnosis for the first time^2,3^. According to its pathogenesis, FSGS can be divided into primary and secondary (podocyte-associated protein mutation, viral infection, drug action, etc.). In 2004, the International Renal Pathology Organization published the FSGS pathological diagnosis and classification criteria (Columbia classification criteria), which can be divided into five types according to the characteristics of lesions: collapsing, tip lesion, perihilar variant, cellular, and not otherwise specified^4,5^. However, the relationship between different etiologies and glomerular lesions, particularly disease activity, has not been clarified. In addition, few studies have evaluated the proportion of segmental sclerosing glomeruli in renal biopsy specimens for the clinicopathological diagnosis and prognostic value of FSGS. Therefore, we conducted a retrospective cohort study to investigate the clinical and pathological findings and prognosis of patients with FSGS with different percentages of glomerular segmental sclerosis.

## Methods

### Patients and research design

**Figure 1:**
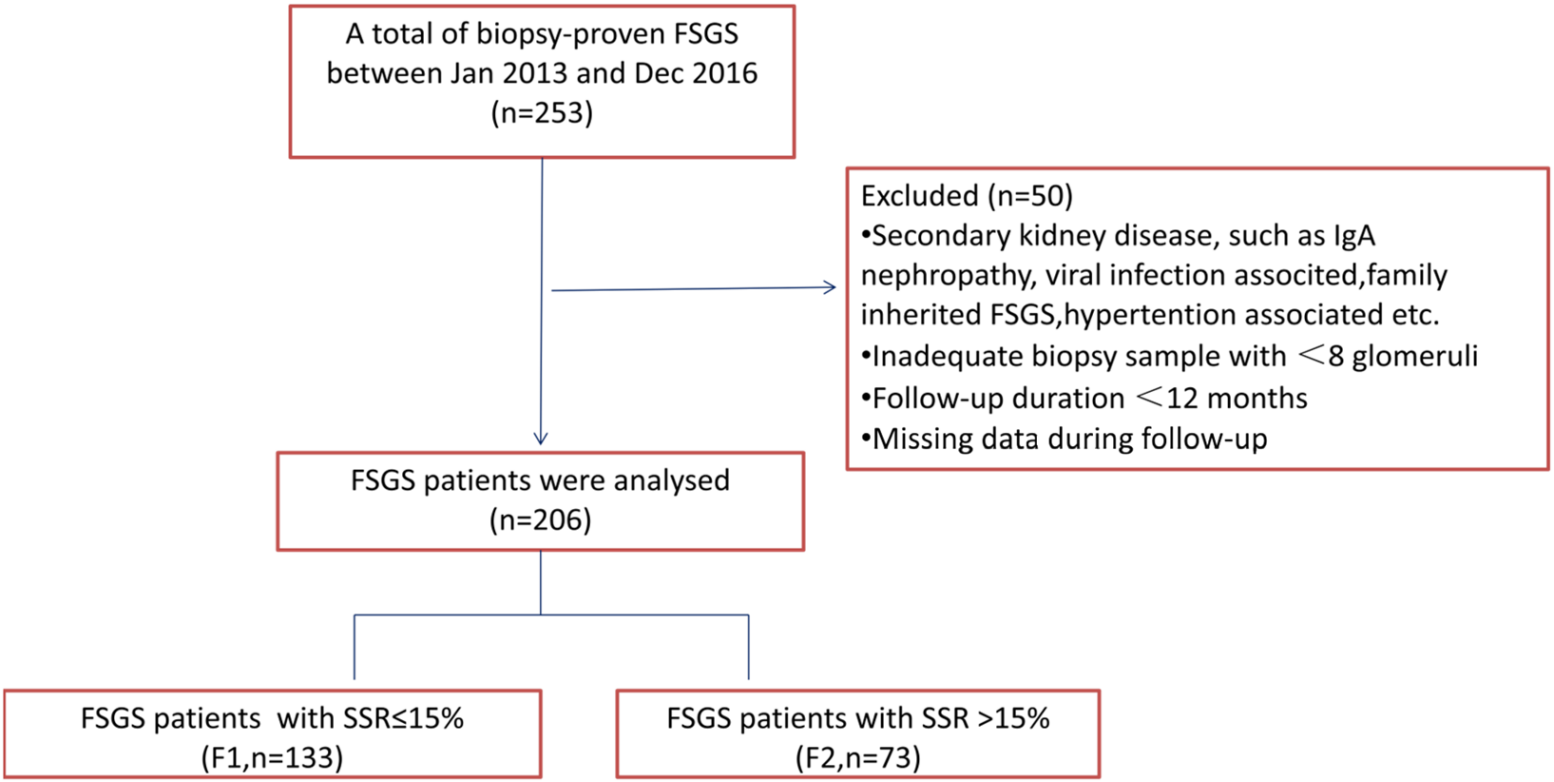
A flow diagram of the study. IgA, immunoglobulin A; FSGS, focal segmental glomerular sclerosis; SSR, segmental glomerular sclerosis ratio.

This single-center retrospective study included 206 patients with FSGS confirmed by biopsy between Jan 2013 and Dec 2016 who were admitted to our hospital (Fig. 1). The inclusion criteria were as follows: (1) patients diagnosed with primary FSGS by renal biopsy and (2) a complete record of clinical history, pathological data (inadequate biopsy sample with ≥ 8 glomeruli), and laboratory results (follow-up duration ≥ 12 months); (3) Patients did not have other primary or secondary kidney diseases, such as IgA nephropathy, viral infection associated, family inherited FSGS, hypertension associated, etc. Baseline demographic and clinical data, including age, sex, systolic blood pressure (SBP), diastolic blood pressure (DBP), serum creatinine (s-Cr), blood urea nitrogen (BUN), uric acid (UA), serum albumin (s-Alb), hemoglobin (Hb), glomerular filtration rate (GFR), urine red blood cells by manual (u-RBC [manual]), urine red blood cells by machine (u-RBC [machine]), and 24 h’ urinary protein excretion (24 h U-Pro), were collected from all patients at the time of renal biopsy. Exclusion criteria were kidney transplantation and other kidney diseases and glomeruli in the glomerulus coexisting with less than 10 glomeruli. In addition, we excluded GFR, blood pressure, and proteinuria data that were missing during the biopsy. GFR was calculated using the Kidney Disease Diet Improvement (MDRD) study equation. Patients of FSGS with NS received glucocorticoids and immunosuppressants. ACEI/ARB was added in patients when their eGFR was greater than 90 ml/min/1.73 m^2^. Patients with isolated proteinuria or isolated hematuria were given immunosuppressants, while glucocorticoids was added if the 24-UP exceeds 3 g/d. Enrolled patients were followed up until December 2020. During the follow-up period, none of the patients received a kidney transplant. The primary outcome was the deterioration of the patient’s condition at the end of follow-up. The evaluation criteria were a combination of GFR (ml/min/1.73 m^2^), which decreased to a permanent value below 50% of the baseline at biopsy, and 24 h U-Pro and u-RBC, which increased to a value 2 times more than the baseline level during the following period. We divided the patients into two groups according to the percentage of segmental sclerosis glomeruli: F1 (SSR (segmental sclerosis rate) ≤ 15%, n = 133) and F2 (SSR > 15%, n = 73).

### Ethics statement

The research protocol and methods were approved by the Ethics Committee of the Second Xiangya Hospital of Central South University (No. 139, Renmin Road, Changsha 410011, Hunan Province, PR China) (Ethical Approval No: Z0636-01). All contents of clinical research were performed in accordance with guidelines and regulations of Ethics Committee of the Second Xiangya Hospital of Central South University for the purpose of protecting the rights and safety of subjects Although this was a retrospective study, in line with the patient’s right to know, all participants were informed of the protocol and provided written consent before participating because the patient’s kidney tissue samples were used for staining and following up on the patient’s prognosis.

### Renal biopsy and pathological classification

All patients underwent ultrasound-guided percutaneous renal biopsy. All biopsy specimens were processed using standard light microscopy (LM), immunofluorescence (IF), and electron microscopy (EM). Renal tissue slides were reviewed and scored by two experienced renal pathologists, and at least 10 glomeruli per slide were required for further review. The degree of mesangial hypercellularity, segmental glomerulosclerosis, tubular atrophy/interstitial fibrosis, and crescentic lesions were evaluated semi-quantitatively for each biopsy specimen. The details of the histological classification were as follows: M1 lesions were defined as mesangial hypercellularity > 50% of glomeruli with ≥ 4 mesangial cells in one or more mesangial areas; T0/T1/T2 is the percentage of tubular atrophy or interstitial fibrosis (< 25%, 25–50%, > 50%); and C0/C1/C2 is the percentage of crescents in the total glomeruli (0, < 25%, > 25%). Also, C4d-0/ C4d-1/C4d-2 is the distribution of C4d positive deposition in glomerulus (0, focal, global). Glomeruli with segmental sclerosis were selected from HE-stained sections of FSGS patients, and we defined the extent of segmental sclerosis as SGR. We calculated the area of SGR in glomeruli using imageJ and then took the mean value of all segmental sclerosis glomeruli as SGR quantification in every patient. The effacement of the foot process was observed in 1–2 glomeruli under EM. Podocyte effacement is defined as normal hiatus membranes between adjacent foot processes disappearing or foot processes disappearing completely. We assessed the length of foot processes in each capillary loop under an EM.

### Statistical analysis

Normally distributed variables were expressed as mean ± SD and compared using a *t*-test or analysis of variance (ANOVA) as needed. Nonparametric variables were expressed as median (interquartile range, IQR) and compared using the Mann–Whitney U test or Kruskal–Wallis test. Categorical variables were expressed as frequencies (percentages) and compared using Pearson’s χ^2^ test or Fisher’s exact test, as appropriate. The Cox survival function was used to assess the association of independent factors with renal outcomes and the presence of segmental sclerosis glomeruli. Cumulative survival was estimated using Kaplan–Meier survival curves. Statistical significance was set at *p* < 0.05. SPSS software (version 17.0) was used to store and analyze the data. All tests were two-tailed.

## Results

### Cohort description

Of the 206 patients, 100 were male (49%), and the mean age was 39.6 ± 15.3 years. The mean SBP was 127.9 ± 18.9 mmHg, and the mean DBP was 82.5 ± 12.3 mmHg. At the time of renal biopsy, patients had a s-Cr of 101.3 ± 81.9 μmol/l and BUN of 6.9 ± 3.9 μmol/l. The UA was 352.9 ± 104.5 μmol/l, and s-Alb was 31.2 ± 12.5 g/dl (Table 1).

**Table 1:** Clinical baseline characteristics of the patients in two groups at renal biopsy.

	Total (n = 206)	F1 (n = 133) SSR ≤ 15%	F2 (n = 73) SSR > 15%	P value
Age (year)	39.6 ± 15.3	41.0 ± 15.1	36.8 ± 15.4	0.057
Sex (male/female)	100/103	65/66	35/38	0.053
Duration (month)	25.2 ± 42.6	26.5 ± 41.9	22.7 ± 44.0	0.538
SP (mm/Hg)	127.9 ± 18.9	126.5 ± 18.7	130.5 ± 19.1	0.150
DP (mm/Hg)	82.5 ± 12.3	82.3 ± 11.5	82.9 ± 13.8	0.750
BUN (mmol/L)	6.9 ± 3.9	6.6 ± 3.4	7.5 ± 4.8	0.148
creatinine (µmol/L)	101.3 ± 81.9	98.0 ± 90.5	107.2 ± 63.7	0.444
eGFR	83.5 ± 37.9	87.1 ± 37.6	76.9 ± 37.9	0.066
UA (µmol/L)	352.9 ± 104.3	341.3 ± 102.2	373.8 ± 106.0	0.034
ALB (g/L)	31.2 ± 12.5	30.9 ± 14.0	31.6 ± 9.1	0.706
Urine protein (g/24 h)	2.7 ± 3.4	2.6 ± 3.5	2.7 ± 3.2	0.937
u-RBC (µ/µl)	154.9 ± 361.1	128.9 ± 240.1	198.7 ± 502.1	0.204
Clinical manifestation				
Chronic renal failure (present/absent)	44/162	24/109	20/53	0.000
Hematuria (present/absent)	145/59	89/42	56/17	0.185
Clinical diagnosis				0.514
Nephrotic syndrome [N (%)]	67 (32.5%)	45 (34.4%)	22 (30.1%)	
Nephritis [N (%)]	117 (56.8%)	72 (55.0%)	45 (61.6%)	
Isolated hematuria [N (%)]	3 (1.5%)	3 (2.3%)	0 (0.0%)	
Isolated proteinuria [N (%)]	17 (8.3%)	11 (8.4%)	6 (8.2%)	
Treatment				
Glucocorticoids [N (%)]	76 (36.9%)	54 (40.6%)	22 (30.1%)	0.323
Immunosuppressants [N (%)]	178 (86.4%)	110 (82.7%)	68 (93.2%)	0.137
ACEI/ARB [N (%)]	78 (37.9%)	60 (45.1%)	18 (24.7%)	0.041

The mean ± standard deviation of normal distribution data was expressed, and t test was used for data analysis; The data of non normal distribution are represented by the median M (1/4, 3/4), and the data are analyzed by Wilcoxon rank sum test. SBP, systolic blood pressure; DBP, diastolic blood pressure; BUN, blood urine nitrogen; CREA, creatine; eGFR, estimated glomerular filtration rate; UA, uric acid; ALB, albumin; u-RBC, urine red blood cell.

### Correlation analysis of clinical and pathological data of FSGS patients

**Figure 2:**
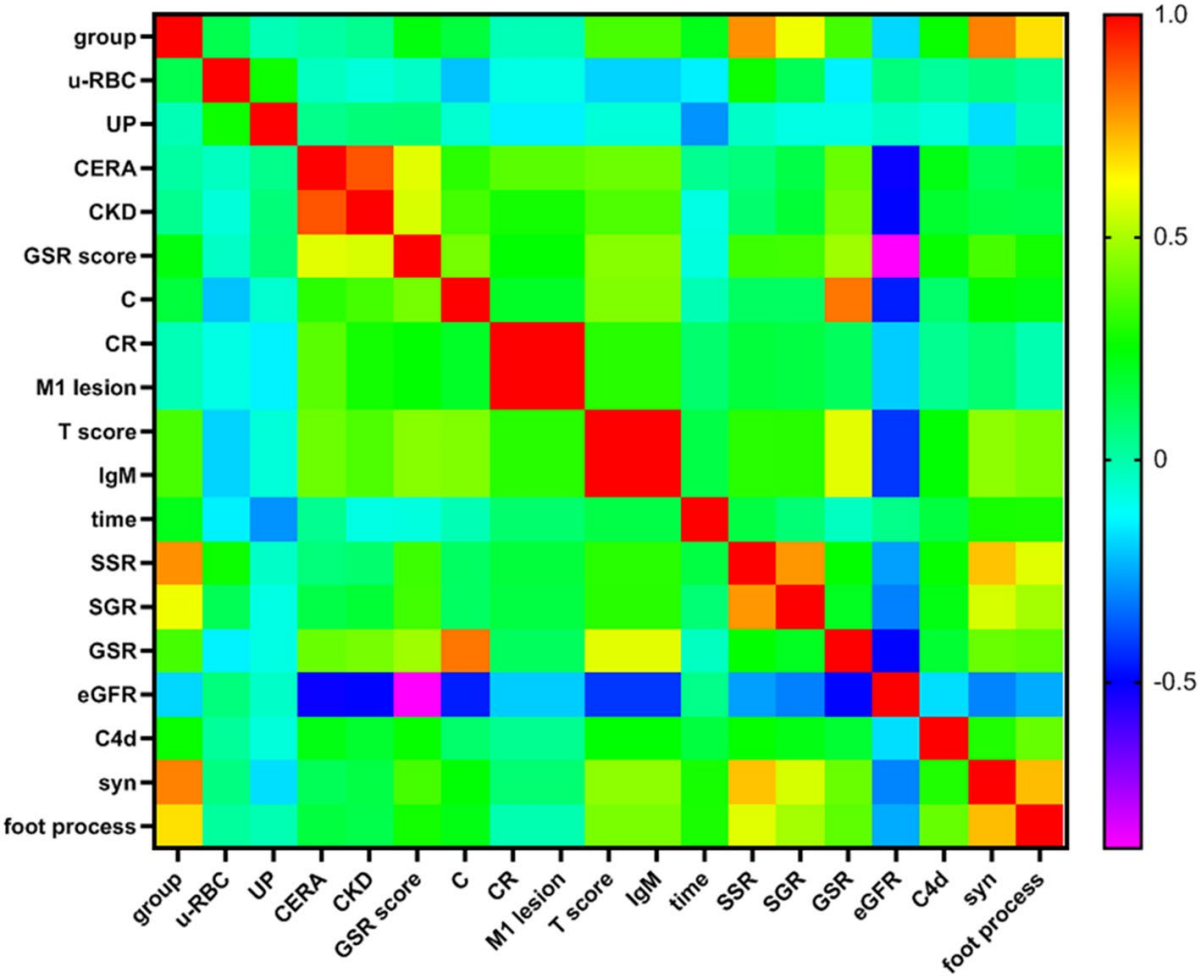
The Pearson correlation analysis between clinical and pathological data of FSGS patients. u-RBC, urine red blood cell; UP, urine protein; CREA, creatine; CKD, chronic kidney disease; GSR, global sclerosis rate; C, crescent; CR, crescent rate; IgM, immunoglobulin M; SSR, segmental sclerosis ratio; SGR, segmental sclerosis in glomeruli; eGFR, estimated glomerular filtration rate; C4d,complement 4d; syn, synaptopodin.

Moreover, we performed a correlation analysis of the clinical and pathological data of patients with FSGS with the baseline data. We demonstrated that there was a significant correlation between the group and u-RBC((urine red blood cell) (r = 0.264, p = 0.018), GSR score (r = 0.338, p = 0.001), T score (r = 0.309, p = 0.003), IgM (r = 0.309, p = 0.003), GSR (r = 0.252, p = 0.015), SGR(r = 0.602, p = 0.000), eGFR (r = − 0.264, p = 0.011), C4d (r = 0.254, p = 0.015), syn (r = 0.712, p = 0.000), and foot process (r = 0.581, p = 0.000) (Fig. 2) (Supplementary Table 1).

### Identification of the optimal classification of SSR

**Figure 3:**
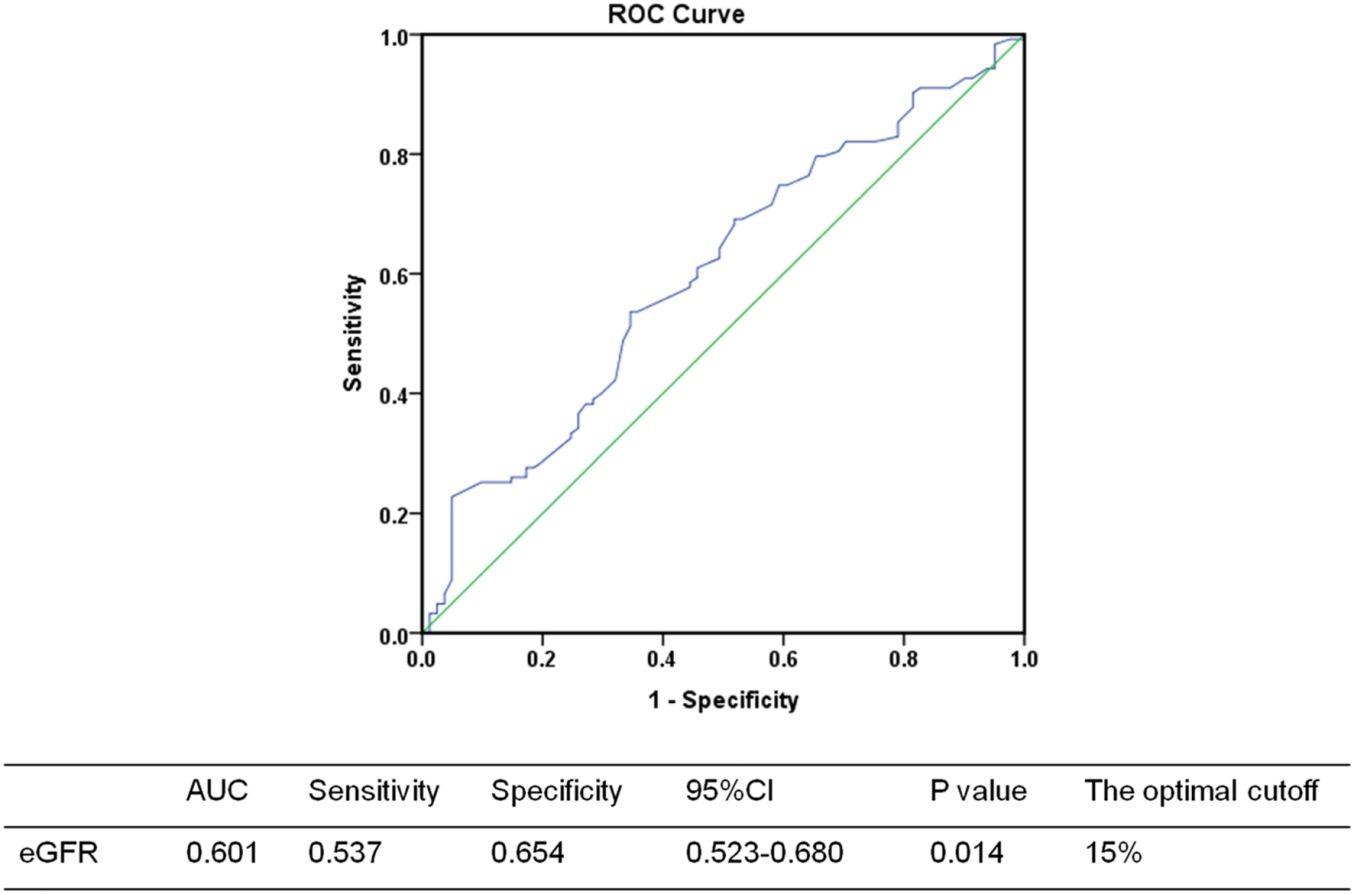
ROC curves of optimal cut-off proportion of SSR in FSGS. AUC: area under curve; CI: confidence interval.

To identify the optimal cut-off, we carried out ROC curve(receiver operator characteristic curve) regarding eGFR decreasing (123 cases positive) (Fig. 3). In the cohort of endpoint events, the optimal cut-off value determined by ROC curves was 15.0% (AUC = 0.601, p < 0.001). Therefore, we divided the cohort into two groups according to SSR: the F1 group (SSR ≤ 15%) and the F2 group (SSR > 15%).

### Clinical manifestations of FSGS patients between the F1 and F2 group during biopsy

The average GFR was 83.5 ± 37.9 ml/min/1.73 m^2^. The u-RBC (manual) and u-RBC (machine) were (8.5 ± 16.8) × 10^5^/ml and 154.9 ± 361.1 µ/µl, respectively. The 24 h U-Pro was 2.7 ± 3.4 g/day. The clinical manifestations of patients in the F1 and F2 groups are shown in Table 1. The F2 group of patients with chronic renal failure was significantly higher than the F1 group (p < 0.01), while the UA level of patients in the F2 group was higher (p = 0.034). Between the two groups, age (p = 0.057), SBP (p = 0.150), DBP (p = 0.750), s-Cr (p = 0.444), BUN (p = 0.148), s-Alb (p = 0.706), u-RBC [manual] (p = 0.509), presence of hematuria, and clinical diagnosis were not statistically significant. There was no difference between the two groups in glucocorticoid and immunosuppressive therapy received, but F1 group had more patients receiving ACEI/ARB therapy.

### Pathological features of FSGS patients between the F1 and F2 group during biopsy

The renal pathological injury score comparison between F1 and F2 patients is shown in Table 2. The two groups had statistically significant differences in the SGR (p = 0.002), IgG (p = 0.042), M1 lesions (p = 0.002) and T score (p = 0.001). Vascular lesions were more severe in the F2 than in the F1 group (p = 0.015). Among the pathological parameters, C score (p = 0.095), IgA (p = 0.276), IgM (p = 0.189), and Fib (p = 0.306) were not statistically significant between the two groups.

**Table 2:** Pathological characteristics of the patients in two groups at renal biopsy.

	Total(n = 206)	F1 (n = 133) SSR ≤ 15%	F2 (n = 73) SSR > 15%	P
Number of glomeruli	15.2 ± 8.2	17.1 ± 8.7	11.7 ± 5.8	0.000
Number of glomerular sclerosis	2.0 ± 2.1	1.9 ± 2.1	2.1 ± 2.2	0.555
Percent of glomerular sclerosis [%,M (1/4, 3/4)]		7.7 (0.0, 16.7)	14.3 (0.0, 28.6)	0.163
Number of focal segmental glomerular sclerosis	2.1 ± 1.5	1.4 ± 0.7	3.2 ± 1.6	0.000
Percent of focal segmental glomerular sclerosis [%,M (1/4, 3/4)]		6.9 (5.0, 8.9)	14.3 (12.5, 20.0)	0.000
SGR (%)	13.0 (8.0, 20.3)	12.5 (7.25, 17.5)	22.0 (20.1, 25.5)	0.002
Number of glomerular adhesion	0.1 ± 0.4	0.2 ± 0.5	0.0 ± 0.2	0.043
Percent of glomerular adhesion [%,M (1/4, 3/4)]		0 (0, 0)	0 (0, 0)	0.624
Number of crescents	0.3 ± 0.9	0.2 ± 0.7	0.4 ± 1.1	0.103
C score [N (%)]				0.095
C1	26 (12.6%)	15 (11.5%)	11 (15.1%)	
C2	4 (2.0%)	1 (0.8%)	3 (4.1%)	0.180
T score [N (%)]				0.001
0	51 (24.8%)	106 (80.9%)	43 (58.9%)	
1	98 (47.6%)	16 (12.2%)	19 (26.0%)	
2	54 (26.2%)	9 (6.9%)	11 (15.1%)	
M1 lesions [N (%)]	86 (41.7%)	45 (34.4%)	41 (56.2)	0.002
Vascular lesions (present/absent)	139 (67.5%)	82/49	57/15	0.015
IgM intensity [N (%)]				
0	150 (72.8%)	95 (72.5%)	55 (76.4%)	0.189
+	33 (16.1%)	18 (13.7%)	110 (13.9%)	
++	18 (8.7%)	17 (13.0%)	3 (4.2%)	
>++	5 (2.4%)	1 (0.8%)	4 (5.6%)	
Other immunoglobulin intensity (0/+/++/>++)				
IgA	164/32/4/3	105/23/3/0	59/9/1/3	0.276
IgG	131/46/21/5	80/34/16/1	51/12/5/4	0.042
C1q	188/9/2/4	122/7/2/0	66/2/0/4	0.106
C3	175/12/11/5	111/10/8/2	64/2/3/3	0.458
Fib	190/7/5/1	125/2/3/1	65/5/2/0	0.306
C4d score [N (%)]				0.004
0	85 (41.3%)	61 (45.8%)	24 (33.0%)	
1	92 (44.7%)	65 (48.9%)	27 (36.9%)	
2	29 (14.0%)	7 (5.3%)	22 (30.1%)	
Synaptopodin intensity	23.2 ± 6.3	27.4 ± 6.7	18.7 ± 5.5	0.000
Foot process effacement (%)	43.1 ± 12.8	29.6 ± 16.3	60.4 ± 17.1	0.000

C, crescent; IgM, immunoglobulin M; IgA, immunoglobulin A; IgG, immunoglobulin G; C4d, complement 4d; C3, complement 3; C1q, complement 1q; Fib, fibronectin SGR, segmental sclerosis in glomeruli.

### The presence of complement and podocyte injury between two groups of the FSGS patients

**Figure 4:**
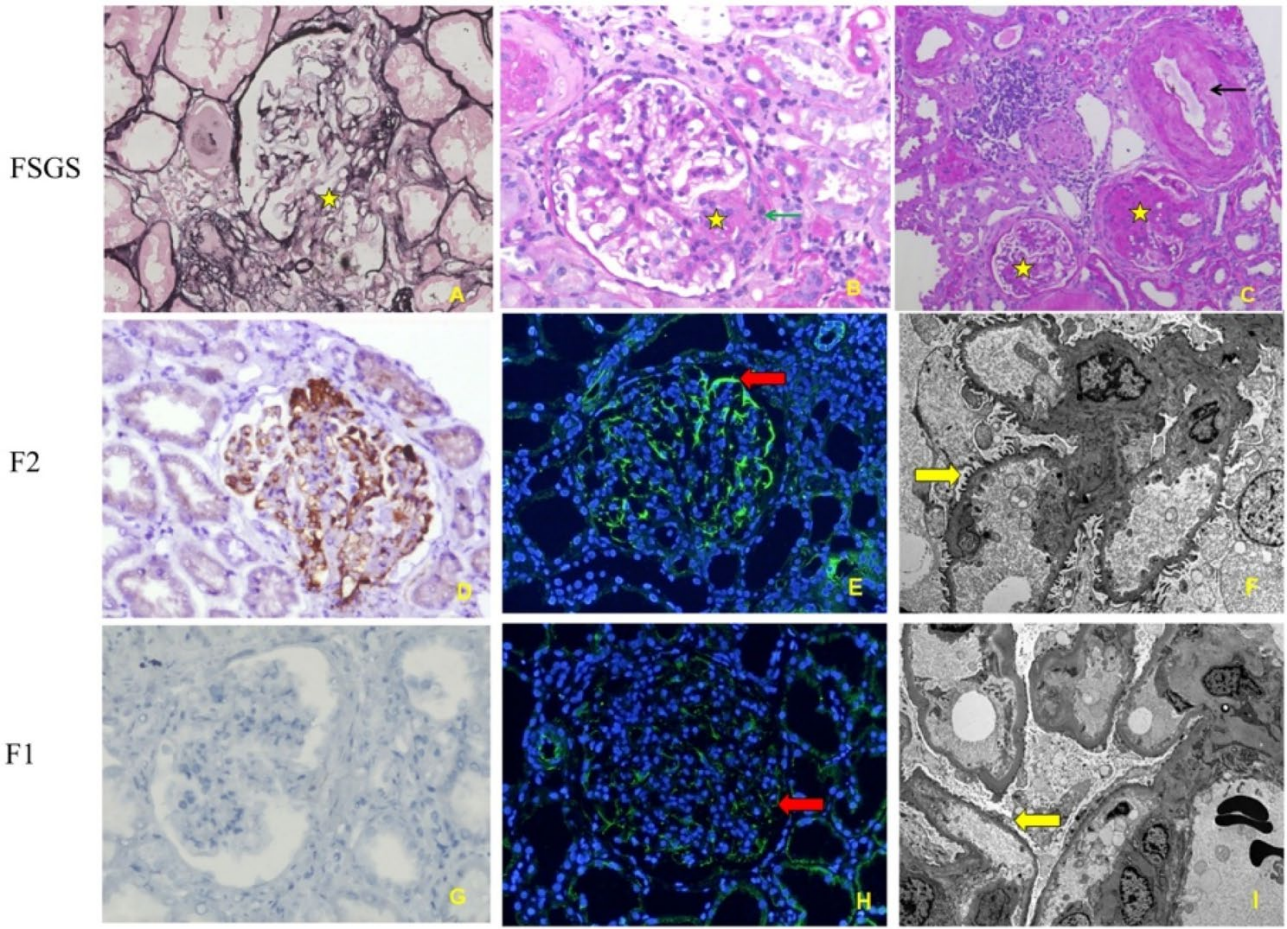
The morphological changes and podocyte lesions of FSGS. The morphological changes of FSGS: (**A**) periodic acid-silver methenamine stain; (**B**) and (**C**) Hematoxylin and Eosin stain; The complement 4d stain: (**D**) and (**G**) the podocyte lesions of FSGS between F1 and F2: synaptopodin (**E** and **H**, the red arrow showed the podocyte), electronic microscopy (**F** and **I**, the yellow arrow showed the footprocess effacement).

We performed immunopathological staining of C4d and synaptopodin in the F1 and F2 groups (Fig. 4). We found that the intensity of synaptopodin in the kidney tissue of patients in the F1 group was higher than that in the other group. Moreover, the percent of C4d score 2 in patients is much more in the F2 group than F1 group (p = 0.004). In addition, the effacement of the foot process was more severe. However, C1q (p = 0.106), and C3 (p = 0.458) levels were not significantly different between the two groups (Table 2).

### Binary logistic regression of clinical and pathological parameters of FSGS patients with different percentages of segmental sclerosis in biopsy

Two-variable logistic regression showed a correlation between the percentage of glomerular segmental sclerosis in the biopsy and the clinical and pathological scores. The group (r = 6.009, p = 0.001), s-Cr level (r = 1.009, p = 0.008), SSR (r = 1.081, p = 0.001), and foot process effacement (r = 1.220, p = 0.003) were positively correlated with the degree of glomerular sclerosis in univariatel logistic regression analysis (Table 3). Then, we added these parameters in a multivariable logistic regression and found that there were significant differences between the different percentages of glomerular segmental sclerosis with group (r = 1.190, p = 0.032), s-Cr level (r = 1.008, p = 0.023) (Table 3).

**Table 3:** Univariate and multivariable logistic regression analysis for segmental glomerular sclerosis according to the clinical parameters and lesion in FSGS patients.

Variables	Univariate analysis		Multivariate analysis	
	Odds ratio	P-value	Odds ratio	P-value
Group 2–1	6.509	0.001	1.190	0.032
Clinical parameters at biopsy				
Gender	2.224	0.121		
Age	0.997	0.867		
Course of disease	0.996	0.521		
u-RBC	1.000	0.561		
UP	1.000	0.252		
Creatinine	1.009	0.008	1.008	0.023
CKD stage				
CKD2	0.500	0.661		
CKD3	1.500	0.810		
CKD4	1.000	1.000		
Histology lesions				
GSR	0.236	0.074		
SSR	1.081	0.001		
CR	1.519	0.206		
SGR	0.993	0.827		
Synaptopodin intensity	1.285	0.466		
Foot process effacement	1.220	0.003	1.706	0.155
C score				
C1	0.135	0.110		
C2	0.063	0.087		
T score				
T1	0.000	0.998	0.565	0.607
T2	0.091	0.011	4.078	0.258
C4d score				
C4d-1	0.952	0.683		
C4d-2	5.830	0.098		
Treatment				
Glucocorticoids	0.233	0.030	0.027	0.152
Immunosuppressants	0.326	0.300		
ACEI/ARB	4.048	0.037	4.630	0.093

CI, confidence interval. u-RBC, urine red blood cell; UP, urine protein; CKD, chronic kidney disease; GSR, glomerular sclerosis rate; SSR, segmental glomerular sclerosis rate; C, crescent; C4d, complement 4d; SGR, segmental sclerosis in glomeruli.

### Kaplan–Meier survival analysis and Cox survival analysis between two groups of FSGS patients

**Figure 5:**
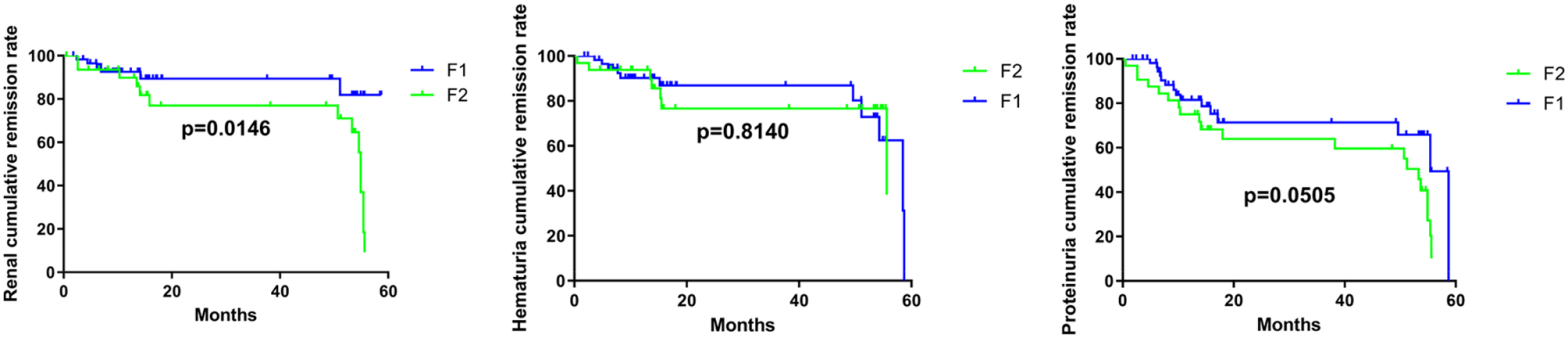
Kaplan–Meier survival analysis between two groups of FSGS patients. The cumulative remission rate of kidney (**A**), hematuria (**B**) and proteinuria (**C**) in FSGS patients during the follow-up.

A Kaplan–Meier survival analysis was performed for the two groups separated based on the percentage of glomerular segmental sclerosis (Fig. 5). The cumulative survival rate of the F2 group was significantly lower than that of the F1 group (p = 0.015). Moreover, the cumulative proteinuria remission rate in the F1 group was significantly higher than that in the F2 group (p = 0.05). However, there were no significant differences in the hematuria cumulative remission rate between the two groups (p = 0.081).

Additionally, we performed a Cox survival analysis (Table 4), which revealed that the percentage of glomerular segmental sclerosis (r = 2.306, p = 0.044), GSR > 20% (r = 4.627, p = 0.005) and the baseline s-Cr at the time of biopsy were related to the development of adverse outcomes.

**Table 4:** Univariate and multivariate Cox survival analysis between two groups in FSGS patients.

		Univariate analysis		Multivariate analysis	
		HR (95% CI)	P-value	HR (95% CI)	P-value
F2		2.78 (1.30–5.93)	0.008	2.306 (1.022–5.207)	0.044
URBC (µ/µl)					
26–50		0.25 (0.08–0.81)	0.020		
51–100		0.52 (0.21–1.26)	0.149		
> 100		0.30 (0.09–0.94)	0.039		
Creatinine (µmol/l)					
121–200		3.023 (1.26–7.29)	0.014	2.85 (1.01–3.55)	0.045
201–300		9.16 (2.54–32.99)	0.001	15.85 (3.55–52.87)	0.022
> 300		2.76 (0.62–12.20)	0.181	6.75 (2.10–35.12)	0.035
CKD stage					
CKD2		3.04 (1.03–8.93)	0.044		
CKD3		3.65 (1.22–10.92)	0.021		
CKD4		3.84 (1.02–14.44)	0.047		
CKD5		7.96 (1.86–34.19)	0.005		
GSR					
< 10%		0.17 (0.02–1.39)	0.098		
19–20%		1.22 (0.35–4.22)	0.753		
> 20%		2.22 (0.93–5.29)	0.072		
T score					
T1		1.99 (0.91–4.35)	0.087		
T2		4.91 (1.74–13.84)	0.003		
Treatment					
Glucocorticoids		0.477 (0.277–0.822)	0.008		

CI, confidence interval; u-RBC, urine red blood cell; CKD, chronic kidney disease; GSR, glomerular sclerosis ratio.

## Discussion

FSGS is a chronic progressive disease that occurs at a high rate in patients with NS. In addition, it has a high recurrence rate after kidney transplantation^6^. In recent years, the incidence of FSGS has increased, hormone therapy is not sensitive, and the prognosis is relatively poor. Approximately 50% of patients gradually progress to CKD within 5–10 years^7^. Glomerulosclerosis is defined as the increase of the mesangial matrix, the disappearance of capillary loops, and the hyaline degeneration of affected segments. According to the distribution, it can be divided into diffuse (sclerotic glomerulus ≥ 50% of all glomeruli in the sample are diffuse), focal (sclerotic glomerulus < 50% of all glomeruli in the sample), spherical (sclerotic glomerulus ≥ 50% of glomerular capillary loops), and segmental (sclerotic glomerulus < 50% of glomerular capillary loops are spherical). In our FSGS cohort, the total SGR was 13%, and the SGR was higher in the F2 group (22.0%) than that in the F1 group (12.5%), which, combined with the reduced podocyte density and the degree of foot process effacement, suggested that the glomerular injury was more severe in the F2 group. FSGS is characterized by focal and segmental glomerulosclerotic lesions under LM. According to the Colombian classification criteria, FSGS is divided into non-specific, perihilar, cellular, tip, and collapse types^4,5^. Matsusaka et al. constructed a FSGS mouse model (NEP25), and the amount of urine protein was positively correlated with the intervention factors in a dose- and time-dependent manner. They also found that podocyte damage can be transmitted to adjacent podocytes to form a vicious circle, segmental sclerosis spreads from one glomerulus to another, nephrons are gradually lost, and residual glomerular compensatory hypertrophy and pathological changes become progressively worse^8^. However, few studies have investigated the proportion of segmental sclerosis glomeruli in renal biopsy specimens. In this study, we focused on the glomerular proportion of segmental sclerosis; we performed a statistical analysis of its correlation with clinical symptoms and prognosis to provide a more intuitive and quantitative theoretical basis for the treatment and outcome of FSGS.

Podocytes, a type of visceral epithelial cell, are inherent cells of the kidney. These are located in the outermost layer of the glomerular filtration membrane. Podocytes, which play a vital role in the renal system, have multiple functions, such as participating in selective glomerular filtration, biosynthesis, and maintenance of the glomerular capillary structure. Podocytes are terminally differentiated cells; therefore, their ability to divide and proliferate is limited. In 2002, Pollak first proposed the concept of podocytopathy, which is a group of glomerular diseases characterized by a reduction in the number and/or density of glomerular podocytes, thickening of the glomerular basement membrane, changes in the composition of the glomerular matrix, foot process fusion, etc.^9^. FSGS is the most common type of podocytopathy disorder. The damage or loss of podocytes plays a crucial role in the occurrence and development of FSGS. We observed that the fluorescence intensity of synaptopodin was significantly reduced in patients with FSGS with a higher proportion of glomerular segmental sclerosis. The decreased intensity of synaptopodin, a specific marker of podocytes^10^, suggested a much greater reduction in the density and loss of podocytes; moreover, FSGS was more severely damaged. We also observed that foot process fusion was more evident in the F2 group under EM. Foot process fusion is a characteristic ultrastructural change in podocyte injury, and the foot processes became flat, fused, and almost disappeared under EM (Electron Microscopy). Foot process fusion and podocyte loss suggest more severe damage to the podocytes. Our analysis indicated that podocyte damage severity was closely related to the proportion of glomerular segmental sclerosis in FSGS.

FSGS lesions not only show structural and functional disorders of podocytes but are also often accompanied by damage to other intrinsic glomerular cells, such as mesangial cells and endothelial cells. Therefore, we added the analysis of other pathological changes in FSGS and found significant differences between the two groups in the degree of mesangial cell proliferation, vascular injury, and renal tubulointerstitial injury. At the same time, dual-factor correlation analysis showed that the intensity of IgM deposition, proportion of glomerular sclerosis, and proportion of renal tubulointerstitial injury were significantly related to the proportion of glomerular segmental sclerosis. Some scholars have developed a podocyte-deficient mouse model for studying FSGS in a podocyte-specific sialylation-deficient mouse model and observed the kidney tissue of mice under LM. Podocyte damage leads to severe glomerular lesions, including mesangial cell proliferation and adhesion^11^. In 2022, Jarcy Zee et al.^12^ applied 48 quantifying histological and 20 ultrastructural indicators to the NEPTUNE (Nephrotic Syndrome Study Network) digital pathology scoring system to diagnose and evaluate MCD (minimal change disease) and FSGS. Through model prediction analysis, they found that glomerulosclerosis was one of the most predictive factors. A comparative study of 207 FSGS patients from three different cohorts found that glomerulosclerosis beyond age adjustment was associated with prognosis. The Cox proportional hazard model showed that FSGS patients with glomerulosclerosis had a 3.2-fold risk of ESRD^13^.

The renal tubules are mainly responsible for recollection, excretion, and concentration functions and maintain the balance of liquids, electrolytes, and acid–bases. We studied 206 patients with FSGS; IFTA (renal tubule/interstitial score) 0/1/2:149/35/22 (72%, 17%, and 11%), and the overall distribution trend of IFTA injuries was similar to that of Ossareh^14^; 71/24/5 (71%, 24%, and 5%). However, the renal tubule atrophy/interstitial fibrosis scores between the two groups differed significantly according to the proportion of segmental sclerotic glomeruli. The dual-factor correlation analysis also confirmed that there was a significant correlation between them, indicating that the FSGS subgroup could better quantify renal lesions. Ossareh et al. also found that the IFTA was a predictor of ESRD in a predictive model designed for the prognosis of FSGS. The risk of ESRD increased 1.06 times with an increase in tubule atrophy and interstitial fibrosis^14^.

Complement fragment C4d is an inert product produced by the cleavage of iC4b in the process of complement activation, which can occur during the activation of the classical complement and mannose lectin pathways. Complement C4, which is activated through the classical pathway, gradually splits into C4d, which can be used as a marker of humoral immune responses^15^. Originally, C4d was mainly used in follicular lymphoma and organ transplantation antibody-mediated rejection. In recent years, C4d has been found to be strongly positive in immune complex-mediated glomerulonephritis, such as thrombotic microangiopathy, lupus nephritis, and membranous nephropathy. In IgA nephropathy (38%) and FSGS, positive expression of C4d was distributed to different degrees^16^. Some studies showed that C4d could be an important indicator of poor prognosis in patients with IgA nephropathy. It was reported that C4d in FSGS could be deposited linearly along the basement membrane in segmental sclerosis through clumps in the mesangial area and coarse granular shapes in vascular poles. The shape and position of C4d deposition in FSGS can not only be used as an effective activity indicator but also as an important indicator of disease prognosis^17^. It was reported that the deposition of C4d in the mesangial region was an independent factor for predicting disease progression and efficacy in FSGS patients^18^. Our study found that C4d is closely related to the proportion of glomerular segmental sclerosis in FSGS.

FSGS is a common pathological type of NS. Damage to podocytes results in injury to the filter barrier, and a large amount of protein leaks out, often leading to hypoalbuminemia and severe proteinuria. Proteinuria remission is an important indicator of the therapeutic effects of nephropathy. Thomas et al. found that the renal survival rate of patients with complete or partial remission of proteinuria was significantly higher than that of patients without remission when they conducted a prognostic analysis of 60 children with FSGS from the glomerular disease collaboration network; complete remission of albuminuria was associated with a 90% risk reduction of ESRD^19^. Prognostic analysis of 116 adult FSGS patients showed that due to the use of immunosuppressants, severe proteinuria at baseline did not affect the prognosis; however, remission of proteinuria was still an independent predictor of renal survival, and even patients with partial remission of proteinuria had a better prognosis^20^. In this study, we analyzed the effect of the FSGS subgroup on proteinuria remission and found that although there was no difference in the baseline proteinuria values between groups, the F1 group was higher than the cumulative remission rate of proteinuria in the other group. The prognosis of FSGS was influenced by many factors^21^. The remission of proteinuria suggested that renal damage was reduced, and renal outcomes were improved^22^. In 2012, some scholars conducted a cross-sectional study on the correlation between globular sclerosis, segmental sclerosis, and serum creatinine levels and clearance rate in fifty patients. They found that in addition to the degree of interstitial fibrosis, serum creatinine levels and clearance rates were closely related to the proportion of segmental sclerosis and global sclerotic glomeruli^23^. This finding is consistent with the results of our study. We further analyzed the survival of the two subgroups of FSGS, and the results showed a significant difference in renal survival rate between the two groups. The Cox multifactor correlation analysis showed that the risk of patients in the F2 group entering ESRD was 2.3 times and the GSR > 20% was 4.6 times higher than that in the F1 group, and the increased serum creatinine value at different levels was also a high-risk factor for FSGS patients to progress to ESRD. Ossareh et al. also reached a consistent conclusion on 201 patients with FSGS using the model to predict the prognosis^14^.

There are some limitations in this study: as a single-center, retrospective observational study, the deviation error of test results increased and a vastly expanded and diverse cohort would be recruited in the future.

## Conclusion

In conclusion, the clinical manifestations, pathological changes, and prognoses of patients with involved glomeruli > 15% are more severe than those with involved glomeruli ≤ 15% in FSGS. Whether or not and when to enhance immunosuppressive therapy remains a difficult point. In the future, large samples, multi-regions, multicenter, and clinical studies will be needed to clarify the predictive factors of FSGS.

### Supplementary Information

Supplementary Table 1.

## Data Availability

All data generated or analysed during this study are included in this published article and its supplementary information files.
