# Author Correction: A new index for the outcome of focal segmental glomerulosclerosis

**DOI:** 10.1038/s41598-024-62818-1

**Published:** 2024-05-24

**Authors:** Liu Chan, Yang Danyi, Chao Chen

**Affiliations:** 1 Department of Nephrology, The Second Xiangya Hospital, Central South University, No.139, Renmin Road, Changsha, 410011 Hunan People’s Republic of China; 2 Hunan Key Laboratory of Kidney Disease and Blood, The Second Xiangya Hospital, Central South University, No.139, Renmin Road, Changsha, 410011 Hunan People’s Republic of China; 3 International Medical Department, The Second Xiangya Hospital, Central South University, Changsha, Hunan People’s Republic of China; 4 National Clinical Research Center for Metabolic Diseases, Key Laboratory of Diabetes Immunology, Ministry of Education, and Department of Metabolism and Endocrinology, The Second Xiangya Hospital of Central South University, Changsha, 410011 Hunan People’s Republic of China

Correction to: *Scientific Reports* 10.1038/s41598-024-59007-5, published online 09 April 2024

The original version of this Article contained an error in the spelling of the author Chao Chen, which was incorrectly given as Chao Cheng.

The original Article has been corrected.

