# Supplementary material for: A new index for the outcome of focal segmental glomerulosclerosis
Source: Sci Rep. 2024 Apr 9;14:8278. doi: 10.1038/s41598-024-59007-5 (PMC11004142; doi:10.1038/s41598-024-59007-5)
Supplement: Supplementary file 1 — Supplementary Table 1. [file 41598_2024_59007_MOESM1_ESM.docx]

**Supplement Table 1. The Pearson correlation analysis between clinical and pathological data of FSGS patients**

|  | **Group** | **u-RBC** | **UP** | **CREA** | **CKD** | **GSR**  **score** | **C** | **CR** | **M lesion** | **T lesion** | **IgM** | **time** | **SSR** | **GSR** | **SGR** | **eGFR** | **C4d** | **syn** | **foot** **process** |
| --- | --- | --- | --- | --- | --- | --- | --- | --- | --- | --- | --- | --- | --- | --- | --- | --- | --- | --- | --- |
| **Group** | **1.000** | **0.264** | **-0.043** | **0.069** | **0.090** | **0.338** | **0.106** | **0.163** | **0.163** | **0.309** | **0.309** | **0.152** | **1.000** | **0.252** | **0.602** | **-0.264** | **0.254** | **0.712** | **0.581** |
| **P** | **0.000** | **0.018** | **0.692** | **0.515** | **0.393** | **0.001** | **0.314** | **0.120** | **0.120** | **0.003** | **0.003** | **0.151** | **0.000** | **0.015** | **0.000** | **0.011** | **0.015** | **0.000** | **0.000** |
| **FSGS%** | **0.785** | **0.133** | **-0.017** | **0.012** | **0.040** | **0.229** | **0.162** | **-0.019** | **-0.019** | **0.354** | **0.354** | **0.211** | **0.785** | **0.352** | **0.777** | **-0.182** | **0.260** | **0.808** | **0.670** |
| **P** | **0.000** | **0.239** | **0.877** | **0.912** | **0.708** | **0.028** | **0.123** | **0.856** | **0.856** | **0.001** | **0.001** | **0.044** | **0.000** | **0.001** | **0.000** | **0.083** | **0.012** | **0.000** | **0.000** |

u-RBC,urine red blood cell;UP,urine protein; CERA,creatine;CKD, chronic kidney disease;GSR, glomerular sclerosis rate; SGR, segmental sclerosis in glomeruli;eGFR,estimated glomerular filtration rate;C, crescents;CR,crescents rate;IgM,immunoglobulin M;SSR, segmental sclerosis rate;C4d,complement 4d;syn, synaptopdin.
